# A high efficiency precision genome editing method with CRISPR in iPSCs

**DOI:** 10.1038/s41598-024-60766-4

**Published:** 2024-04-30

**Authors:** Avinash Singh, G. Dalton Smedley, Jamee-Grace Rose, Kristina Fredriksen, Ying Zhang, Ling Li, Shauna H. Yuan

**Affiliations:** 1 Department of Neurology, University of Minnesota, Twin Cities, Minneapolis, MN USA; 2 Graduate Program in Neuroscience, University of Minnesota, Twin Cities, Minneapolis, MN USA; 3 Minnesota Supercomputing Institute, University of Minnesota, Twin Cities, Minneapolis, MN USA; 4 Department of Experimental and Clinical Pharmacology, University of Minnesota, Twin Cities, Minneapolis, MN USA; 5 Minneapolis Veterans Administration Health Care System, Minneapolis, MN USA

**Keywords:** CRISPR, iPSC, Gene editing, High efficiency, Single nucleotide polymorphism, Cell biology, Stem cells, Neurology

## Abstract

The use of genetic engineering to generate point mutations in induced pluripotent stem cells (iPSCs) is essential for studying a specific genetic effect in an isogenic background. We demonstrate that a combination of p53 inhibition and pro-survival small molecules achieves a homologous recombination rate higher than 90% using Clustered Regularly Interspaced Short Palindromic Repeats (CRISPR) in human iPSCs. Our protocol reduces the effort and time required to create isogenic lines.

## Introduction

Genome editing is one of the most powerful tools being developed in the field of biotechnology. The ability to alter an organism's genome enables scientists to investigate challenging evolutionary and medicinal issues in more intricate systems^1^. Genome-Wide Association Studies (GWAS) have yielded a large number of single nucleotide polymorphisms (SNPs), genetic variants associated with increased risk of disease^2^. Gene editing in iPSC lines allows production of genetically engineered isogenic lines containing the SNPs, and these cells may illustrate how such genetic mutations can cause disease. The level of editing varies from theoretically simple gene knockouts to more complicated edits, such as full gene insertions or point mutations. While genomic editing technology has rapidly advanced, several challenges remain unresolved^3–5^.

Clustered Regularly Interspaced Short Palindromic Repeats (CRISPR) has become the preferred method for genome editing in both plant and animal models^6^. Current methods typically use the nuclease variants Cas9 and Cas12a^6^. The CRISPR-Cas9 and -Cas12a systems interact with a guide RNA (gRNA) that consists of two segments: a CRISPR RNA (crRNA) that specifies the genomic target site and a transactivating CRISPR RNA (tracrRNA) that directly binds with Cas nuclease to form ribonucleoprotein (RNP)^7^. The innovative CRISPR-Cas gene edit technology is simpler and more affordable to configure compared to prior gene altering techniques, leading to an extensive utilization^2,8,9^. However, despite significant progress in the CRISPR-Cas editing system, further optimization to improve the efficiency and the percentage of successful edits is urgently needed especially whenever more than one risk variant is associated with a gene of interest, requiring the generation of multiple lines with different SNPs through knocking in multiple genetic modifications sequentially.

In this study, we aimed to develop a highly efficient and easily adaptable gene editing protocol to overcome the obstacles that currently limit the efficiency of point mutation in cell lines. There is significant cell death noted due to double-stranded chromosomal break associated with CRISPR and single cell cloning^10^. On the other hand, inhibition of Rho- related kinase (ROCK) or p53 pathway has been reported to improve editing efficiency by preventing cell death^8^. We hypothesized we could improve cell recovery through the inhibition of p53 activation and thus increase editing efficiency.

## Results

**Figure 1:**
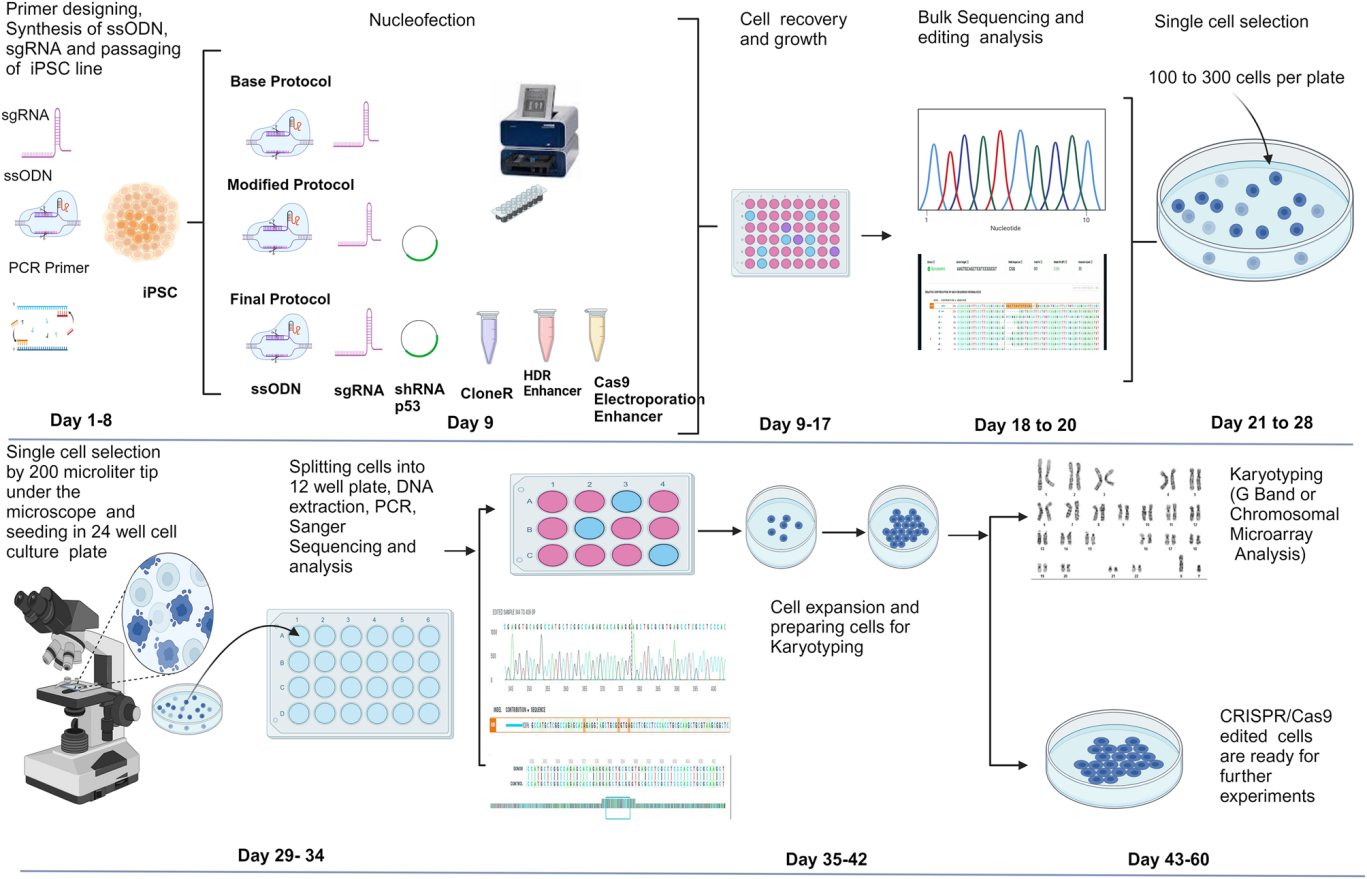
Schematic diagram representing outline of study design. Editing was done by using CRISPR-Cas9 technology; base, modified and final protocols. Base protocol is defined as using only gRNA and ssODN. Modified protocol is defined as using shRNAp53 in gRNA and ssODN cocktail. Final protocol is defined by using ssODN, sgRNA (single guide RNA), shRNAp53, electroporation enhancer, CloneR and HDR enhancer.

**Figure 2:**
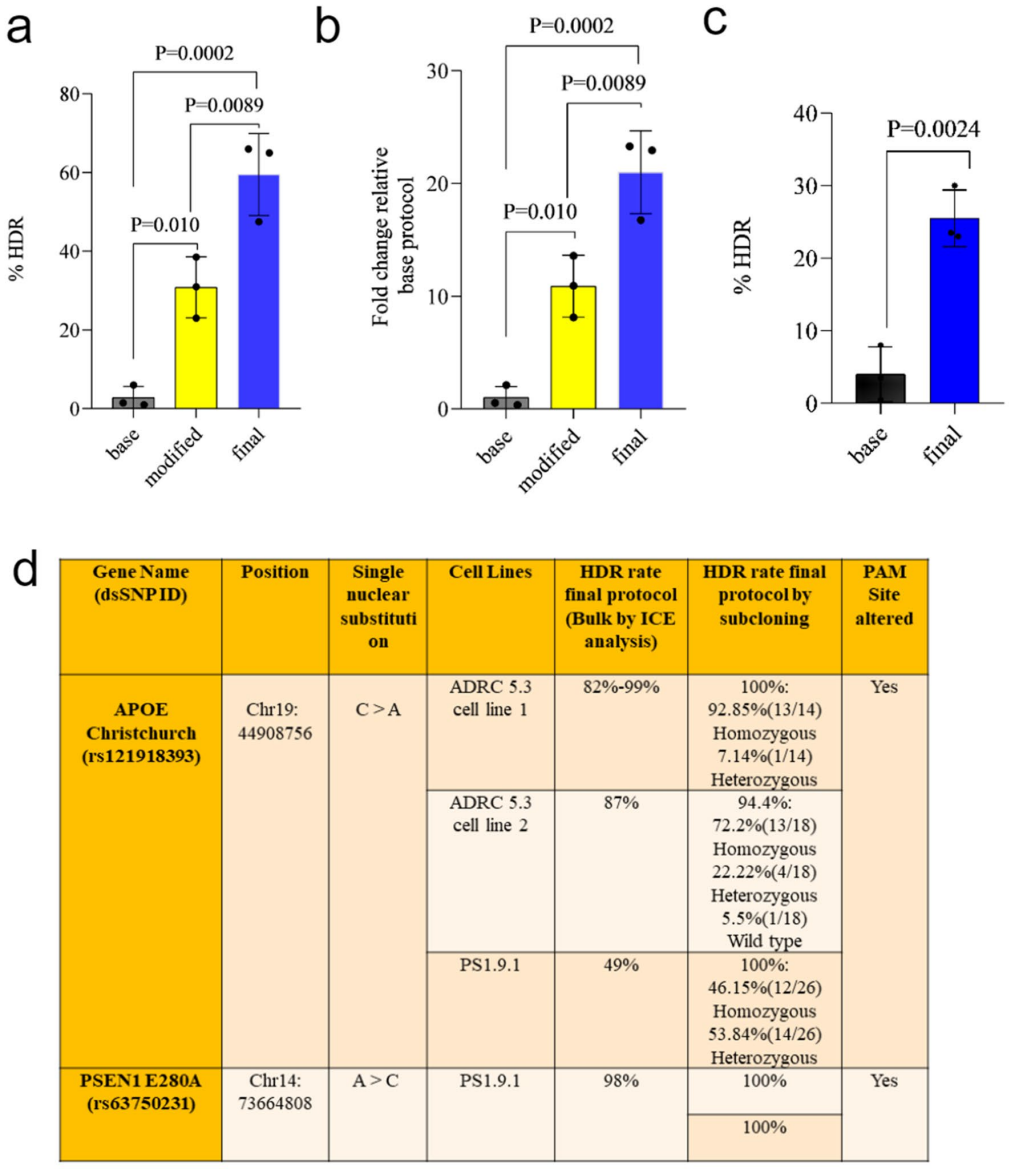
p53 inhibition and pro-survival factors improve editing efficiency (**a**), Percent HDR rate after including shRNAp53 and HDR enhancer, electroporation enhancers and CloneR in the base protocol for knocking in the SNP variant rs867529 in the human *EIF2AK3* gene. (**b**), Fold change relative to base protocol after shRNAp53 and HDR enhancer, electroporation enhancer and CloneR in the base protocol SNP variant rs867529 in the human *EIF2AK3* gene. (**c**), Percent HDR rate increase by including shRNAp53 and HDR enhancer, electroporation enhancer and Clone R in the base protocol in another SNP variant (rs13045) for the *EIF2AK3* gene. n = 3 experiments with 1–3 biological replicates in each experiment. Error bars indicate standard deviation. Statistical analysis was performed by ANOVA with Tukey’s post-hoc analysis in (**a** and **b**), student’s t-test in (**c**). (**d**) Details of cell lines that were edited using the final protocol and their percent HDR rate.

We tested our approach (Fig. 1) on GWAS risk variant associated with tauopathy. The SNP variant rs867529 in the human *EIF2AK3* (Eukaryotic Translation Initiation Factor 2 Alpha Kinase 3) gene results in an amino acid change from serine to cysteine at amino acid position 136, by changing the nucleotide cytosine (C) to guanine (G) (Supplementary Fig. 1a). We identified a Protospacer Adjacent Motif (PAM) four nucleotides from the SNP and directed a DNA cut by Cas9 at the SNP. As previously reported, a homozygous Homology-Directed Recombination (HDR) event is best achieved when the guide RNA induces the cleavage less than ten nucleotides from the intended mutation^11^. We used single strand oligonucleotide (ssODN) as the repair template to introduce the desired editing for the homologous recombination. To avoid re-editing and improve editing efficiency, we introduced a silent mutation to the PAM site in the repair template^11^. We observed transfection with plasmid encoding shRNA (small hairpin RNA) against p53 improved the HDR rate to a mean of 30.8% by ICE (Inference of CRISPR Edits) analysis (Synthego), which was 11 times higher than the base protocol (Fig. 2a, b). When we added an HDR (homology-directed repair) enhancer (IDT), electrophoresis enhancers (IDT) and ​CloneR (STEMCELLTechnologies) to improve the cell survivability, the HDR increased to a mean of 59.5%, 21 times higher than the base protocol (Fig. 2a, b).

We also tested this approach with another SNP variant (rs13045) for the *EIF2AK3* gene, which changes the nucleotide at amino acid position 166 from guanine (G) to adenine (A), resulting in amino acid change from arginine (R) to glutamine (Q). We tested a Cas9 cleavage site which is four nucleotides away from the SNP. Using the base protocol, the HDR efficiency was 4%, but using the optimized final protocol, the HDR efficiency was increased to 25%. Here, we did not alter the PAM site, for alteration would introduce a missense mutation. The increase in HDR efficiency is about sixfold higher compared to the base protocol (Fig. 2c).

Next, we examined the efficiency of our final protocol in other iPSC lines. We applied the final protocol to introduce the *APOE R136S* Christchurch mutation in three different iPSC lines (PS 1.9.1, ADRC iPSC 5.3 cell line 1 and ADRC iPSC 5.3 cell line 2) (Fig. 2d). We observed 49% knock-in efficiency by ICE analysis in bulk sequencing in PS1.9.1 cells, and 100% knock-in in all of the subclones sequenced [(46.15% (12/26) Homozygous, 53.84% (14/26) Heterozygous]. Similarly, we observed 82–99% knock-in efficiency in ADRC 5.3 cell line 1 and 87% knock-in efficiency in ADRC 5.3 cell line 2 by ICE analysis. 100% and 94.4% of the subclones respectively have been edited. We also performed reverse mutation in the PS1.9.1 iPSC line to correct the *PSEN1 E280A* mutation using the final protocol. Comparably to other knock-ins, we observed 97 to 98% knock-in by ICE analysis in bulk sequencing and 100% knock-in after subcloning to single cell clones (Fig. 2d). Importantly, all the clones were found to be karyotypically normal by G-banding analysis (Supplementary Fig. 1b).

We performed whole genome sequencing (WGS) on PS1.9.1 *APOE* Christchurch edited cells to detect the gene editing status and any unwanted off target modifications. The WGS confirms the successful introduction of the Christchurch mutation. Analysis using DELLY^12^ for the detection of somatic large structural variations, revealed an inversion on chromosome7. However, no detectable off-target modifications were noted from the CRISPR Cas9 by using Cas-OFFinder software ^13^ (Supplementary Fig. 1c).

## Discussion

We show that by combining p53 inhibition and pro-survival small molecules, a homologous recombination rate higher than 90% is achievable in multiple genetic loci, iPSC lines and labs. It has been demonstrated that Cas9 induces apoptosis in cells and inhibiting the apoptotic pathway may improve editing efficiency ^14^. Additionally, electroporation can harm the cells and can cause cell death. We show that introduction of p53 shRNA “significantly” improved editing efficiency at all sites. Chemical inhibition of p53 is plausible, but should be empirically tested to determine the effectiveness, for small molecules can be less specific compared to genetic approaches^15^. In addition, we have discovered that the p53 shRNA and additional supplements such as CloneR and ROCK inhibition further improve editing efficiency, possibly by allowing nucleoporated cells to survive. The co-transfection of a plasmid that produced BCL-XL, the anti-apoptotic isoform of the BCL2-like 1 (BCL2L1) gene, and the inhibition of the p53 pathway, have been shown to be associated with an improvement in cell survival^9^. Additionally, it has been demonstrated that a variety of electroporation tools can improve editing efficiency and cell survival^16^. Recently, a cocktail of 4 different compounds has been shown to improve iPSC survival ^17^. We have discovered that using multiple survival-promoting strategies increases our chances of obtaining the desired point mutated clone. The combination of p53 inhibition with pro-survival small molecules may offer a more targeted and synergistic approach to enhance the HDR efficiency. However, the choice between these approaches depends on the specific experimental context, including the cellular model, the nature of DNA repair defects, and the desired outcomes. Utilization of anti-apoptotic agents may raise concerns about promoting chromosome abnormality for growth advantage. However, we have found that karyotyping with G-banding showed the use of anti-apoptotic drugs for short periods of time does not enhance the selection of an abnormal karyotype.

In summary, we found that modification of the CRISPR protocol using a combination of p53 inhibition and pro-survival small molecules to promote cell survival can increase the efficiency of gene editing and reduce the time required to as little as 8 weeks to produce isogenic lines for study as disease models. While there is still variability in the molecular biology of certain variant sites and the long lasting effects of this workflow on the overall cell fitness, these improvements would allow a broader array of researchers, particularly those targeting challenging variants, to develop isogenic lines without the extensive resource and time commitments currently necessary for such work.

## Methods

### iPSC cell culture and maintenance

iPSCs were maintained in Stemflex (Gibco # A334901) and mTeSR Plus (STEMCELL Technology # 100-0276) medium in feeder-free conditions on a basal matrix of Matrigel (Corning # 47743-706). ReLeSR (STEMCELL Technologies # 100-0484) was used for routine maintenance splitting.

### Nucleofection

Nucleofection was performed when cells were at 80–90% confluent in a 6-well culture plate. Cell culture media was changed 1 h prior to nucleofection with cloning media composed of Stemflex with 1% Revitacell (Gibco #A2644501) and 10% CloneR (STEMCELL Technologies #05888). Cells were dissociated with Accutase (VWR # AT104) for 4–5 min. The RNP Complex was prepared by combining 0.6 µM guide RNA (IDT) and 0.85 µg/µL of Alt-R S.p. HiFi Cas9 Nuclease V3 (IDT #108105559) and incubated at room temperature for 20 to 30 min. 0.5 µg pmaxGFP (LONZA #V4XP3032), 5 µM ssODN, and the pre-prepared RNP complex were combined and used in all protocols. 50 ng/µL pCXLE-hOCT3/4-shp53-F (Addgene #27077) plasmid was co-transfected for p53 knockdown in modified protocol. In the final protocol, Alt-R Cas9 Electroporation Enhancer at 1:25 dilution (IDT #1075915) and the pCXLE-hOCT3/4-shp53-F plasmid were also used. In tandem with experimental trials, two external controls were tested simultaneously. A GFP control reaction contained cells and 50 ng/µL pmaxGFP. No pulse control reaction was created with all components. 5 × 10^5^ cells were used per reaction. The 20 µL reaction was transferred to a 16-well nucleocuvette strip (LONZA #V4XP3032) and nucleofected using LONZA 4D Nucleofector on the CA137 pulse program except for the well containing the no pulse control. After nucleofection, the nucleofected cells were kept in incubator for 10 min and then were plated and incubated at 37 °C in a humidified 5% CO_2_ incubator. For the final protocol, cells were incubated in media containing 10% CloneR, 1% Revitacell and Alt-R Cas9 HDR Enhancer (IDT #10007910).

### Design of the Guide RNA and the HDR template

The guide RNA was designed using the IDT Alt-R CRISPR-Cas9 guide RNA. (https://www.idtdna.com/site/order/designtool/index/CRISPR_PREDESIGN). Selection of guide RNA was based on the proximity of the cleavage site and the desired gene editing site. Additional criteria include the highest chance of successful targeting and the least chance of off target editing reported by the program. All the guide RNA that we used in this study in given in Supplementary Table 1. The HDR templates were also designed by using the IDT Alt-R CRISPR HDR Design Tool (https://www.idtdna.com/pages/tools/alt-r-crispr-hdr-design-tool). Details of HDR is provided in Supplementary Table 2.

### Determining homology directed repair (HDR) efficiency

Genomic DNA was extracted using the Quick DNA mini prep kit (Zymo Research #3020). The Q5 High-Fidelity DNA polymerase was used to amplify the region around the editing site (New England Biolabs #M0491L) using forward and reverse primers (Supplementary Table 3). PCR reactions were resolved on a 1% electrophoresis gel and bands of the desired size were excised and purified using a Zymoclean Gel DNA Recovery Kit (Zymo Research #D4001). Samples were then submitted for Sanger sequencing (Eurofins). The results from sequencing were analyzed using Synthego’s ICE analysis software to determine the percentage of HDR^18^ INDELs and wild type cell populations in each sample.

### Karyotyping

Karyotyping was performed using G-banding by the University of Minnesota Cytogenetics Core Laboratory shared services at the Masonic Cancer Genomics Center.

### Whole genome sequencing (WGS)

Genomic DNA was prepared using whole cell lysate from iPSC using QuickDNA microprep kit (Zymo #D3021). WGS was performed using NovaSeq by the University of California, San Diego Institute for Genomic Medicine (IGM) Genomics Center Core. The sequencing data first underwent quality check by FastQC (Babraham Institute). Because each sample was sequenced in two runs, the data was merged for each sample. GATK MuTect2^19^. Genome Analysis Toolkit (GATK) Mark duplicate and GATK Base Quality Recalibration (MIT) were used to remove extract duplicated reads and to refine the Burrows-Wheeler Aligner (BWA) alignment. Variant calling was performed by GATK MuTect2 for somatic SNPS and indels (size <  = 100base pairs) and DELLY^12^ were used to identify somatic mutations and large indels in whole genome sequencing of clone 14 and clone 23 strains using default pipeline parameters. Parent strain PS1.9.1 was used as reference matching-normal dataset in both analyses. Cas-OFFinder^13^ was used to predict off-target sites in human genome (hg38) using the following parameters: CRISPR (RGENs) = 5′-NGG-3′, mutations <  = 2, bulge size = 1. There are total 44 predicted off-target sites, which were manually examined for reads coverage and mutation detection per site.

### Supplementary Information

Supplementary Information.

Supplementary Figure 1.

Supplementary Tables.

## Data Availability

Experimental data is available upon request, subject to UMN institutional guidelines. Material and data requests will be considered based on a proposal review, completion of a material transfer agreement and/or a data use agreement, and in accordance with the UMN intuitional guidelines. Please contact the corresponding author SHY (syuan@umn.edu) for requests.
