# Supplementary material for: A high efficiency precision genome editing method with CRISPR in iPSCs
Source: Sci Rep. 2024 Apr 30;14:9933. doi: 10.1038/s41598-024-60766-4 (PMC11061145; doi:10.1038/s41598-024-60766-4)
Supplement: Supplementary file 1 — Supplementary Information. [file 41598_2024_60766_MOESM1_ESM.docx]

**Supplementary Figure Legend**

**Figure 1**: a, Schematic diagram illustrates the targeting design for single nucleotide substitution from cytosine (C) to guanine (G) resulting in amino acid change from serine to cysteine by CRISPR. The PAM site denoted by a red box is three nucleotides downstream of the SNP site. The red arrow indicates the double stranded bread induced by Cas9. The repair template also introduces a silent mutation in the PAM site to prevent recutting after editing. b, Representative G band karyotyping result of CRISPR edited iPSCs. c, Whole genome sequencing results showing no detectable off target sites among the CRISPR edited PS1.9.1 cells (Clone 23, Clone 14, PS1-10-10).
