# Supplementary figures and images for: A high efficiency precision genome editing method with CRISPR in iPSCs
Source: Sci Rep. 2024 Apr 30;14:9933. doi: 10.1038/s41598-024-60766-4 (PMC11061145; doi:10.1038/s41598-024-60766-4)

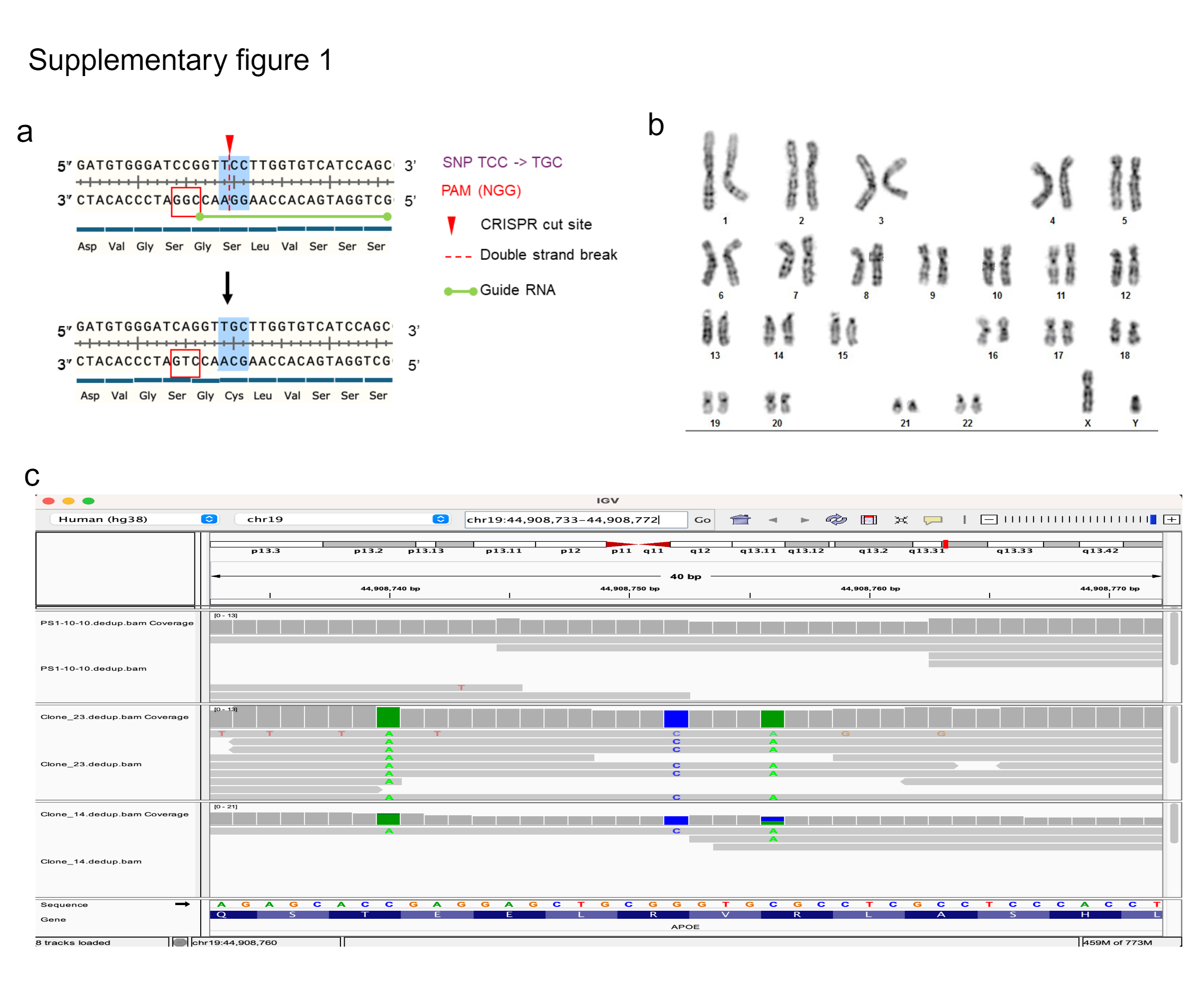

Supplement: Supplementary file 2 — Supplementary Figure 1. [file 41598_2024_60766_MOESM2_ESM.png]
