# Supplementary material for: A high efficiency precision genome editing method with CRISPR in iPSCs
Source: Sci Rep. 2024 Apr 30;14:9933. doi: 10.1038/s41598-024-60766-4 (PMC11061145; doi:10.1038/s41598-024-60766-4)
Supplement: Supplementary file 3 — Supplementary Tables. [file 41598_2024_60766_MOESM3_ESM.docx]

**A high efficiency precision genome editing method with CRISPR in iPSCs**

Avinash Singh^1^, G. Dalton Smedley^1^, Jamee Rose^1^, Kristina Fredriksen^2^, Ying Zhang^3^, Ling Li^2,4^ , Shauna H. Yuan^1,2,5^

1. Department of Neurology, University of Minnesota, Twin Cities, Minneapolis, MN, USA

2. Graduate Program in Neuroscience, University of Minnesota, Twin Cities, Minneapolis, MN, USA

3. Minnesota Supercomputing Institute, University of Minnesota, Twin Cities, Minneapolis, MN, USA

4. Department of Experimental and Clinical Pharmacology, University of Minnesota, Twin Cities, Minneapolis, MN, USA

5. Minneapolis Veterans Administration Health Care System, Minneapolis, MN, USA

**Supplementary Tables**

**Supplementary Table 1. Information of guide RNA used in this study**

| dsSNP ID | gRNA Sequences PAM Site | |
| --- | --- | --- |
| rs867529 | GCTGGATGACACCAAGGAAC TGG |  |
| rs13045 | TTTCACGGTCTCGGTCCCAC CGG |  |
| rs121918393 | GAGGCGCACCCGCAGCTCCT CGG |  |
| rs63750231 | TATGCTGGTTGAAACAGCTC AGG |  |
|  |  |  |

**Supplementary Table 2: Information of HDR templates used in this study**

| dsSNP ID | HDR Template Sequences |
| --- | --- |
| rs867529 | TTAACAGAAAATTCTTACCTCTGGTTTGCTAAGGCTGGATGACACCAAGCAACCTGATCCCACATCCAAATCCCACTGCTTTTTACCATGATTTTCAGGA |
| rs13045 | GTGATTCAACTGTGAAAGGAACTGTTTCCATGCTTTCACGGTCTTGGTCCCACTGAAAGAGGGCTCCATCCAGGGAAGGAATGATCATCTTATTCCCAAA |
| rs121918393 | GGAGCCGCTTACGCAGCTTGCGCAGGTGGGAGGCGAGGCTCACGCGCAGCTCCTCTGTGCTCTGGCCGAGCATGGCCTGCACCTCGCCGCGGTACTGCAC |
| rs63750231 | CAAATACTTACAGGAGTAAATGAGAGCTGGAAAAAGCGTTTCATTTCTCTCTTGAGCTGTTTCAACCAGCATACGAAGTGGACCTTTCGGACACAA |
|  |  |

**Table3: Primes used for on targeting analysis**

| dsSNP ID | Forward primer | Reverse primer |
| --- | --- | --- |
| rs867529 | AGCCCGGTTTCTATGAGAGC | TGGGATTTTGCAGCACAGAT |
| rs13045 | TGGCCCATGGTAAACTGAGT | CGCTTGAACCCACTTGAACC |
| rs1805165 | TCCACGTGAGGTACCTTGTT | GTGGTTGGTCTTGGAGGAGA |
| rs121918393 | TCCTAGCTCCTTCTTCGTCT | CTGCTCCTTCACCTCGTCCA |
| rs63750231 | ATGTTGTCTCCCCCACCCCCAC | CAAAGAGATCTGCAGGAGTTCCAGG |
